# Emerging technologies for early risk stratification and precision management of diabetic kidney disease: a multimodal framework integrating digital phenotypes and clinical biomarkers

**DOI:** 10.3389/fendo.2025.1728293

**Published:** 2026-01-09

**Authors:** Lingdong Meng, Zhen Li, Ling Xu, Fang Wei, Hongyan Ji, Lankun Zhang, Anning Zhu, Zhijia Zhou

**Affiliations:** 1 Hemodialysis Center, Yangzhou Hospital of Traditional Chinese Medicine, Yangzhou, China; 2 Department of Tuina, Yueyang Hospital of Integrated Traditional Chinese and Western Medicine, Shanghai University of Traditional Chinese Medicine, Shanghai, China; 3 Traditional Chinese Medicine Rehabilitation Center, Second Affiliated Hospital of Nanjing University of Chinese Medicine, Nanjing, China; 4 Department of Endocrinology, The Second Affiliated Hospital, School of Medicine, Zhejiang University, Hangzhou, China

**Keywords:** diabetic kidney disease, early risk stratification, digital phenotyping, tongue and pulse analysis, machine learning

## Abstract

**Background:**

Diabetic kidney disease (DKD) is a major microvascular complication of diabetes, often progressing silently and leading to end-stage kidney disease (ESKD) and cardiovascular morbidity. Early identification and risk-adapted intervention are crucial to improving long-term outcomes, yet existing clinical workflows are limited by delayed diagnosis and underutilization of available therapies.

**Methods:**

We propose and evaluate a multimodal, risk-driven framework for the early recognition and individualized management of DKD. The approach integrates: (1) standard renal function metrics—estimated glomerular filtration rate (eGFR) and urine albumin-to-creatinine ratio (uACR)—together with validated prediction models; (2) molecular biomarkers including metabolomics, gut microbiota, and peritoneal dialysis effluent signatures; (3) digital phenotypes derived from standardized acquisition of tongue images and pulse waveforms, rooted in Traditional Chinese Medicine (TCM) diagnostics; and (4) longitudinal data from wearable devices and remote monitoring platforms. Digital features are quantified using image processing and optical signal analysis and incorporated into multimodal prediction models. Treatment is escalated based on risk stratification using renin–angiotensin–aldosterone system (RAAS) inhibitors, sodium–glucose cotransporter 2 (SGLT2) inhibitors, non-steroidal mineralocorticoid receptor antagonists (MRAs), and glucagon-like peptide-1 (GLP-1) receptor agonists. Real-time monitoring of therapeutic efficacy and safety is conducted using process end points such as eGFR slope and uACR trends.

**Results:**

Incorporation of quantifiable tongue and pulse features provides a novel, low-cost, and non-invasive risk enrichment layer that complements biochemical and omics-based markers. Multilayered risk stratification enables earlier identification of fast progressors and more timely treatment intensification. Evidence from landmark trials—including Dapagliflozin and Prevention of Adverse Outcomes in Chronic Kidney Disease (DAPA-CKD), Empagliflozin in Patients with Chronic Kidney Disease (EMPA-KIDNEY), Finerenone in Reducing Kidney Failure and Disease Progression in Diabetic Kidney Disease (FIDELIO-DKD), and Effects of Semaglutide on Chronic Kidney Disease (FLOW)—supports the clinical utility of this approach. A closed-loop monitoring strategy based on process metrics and safety thresholds is proposed. We also outline ethical, regulatory, and data governance considerations necessary for clinical translation.

**Conclusion:**

The integration of traditional clinical markers, digital TCM-derived phenotypes, and multi-omics data represents a promising paradigm for early, personalized, and dynamic DKD care. Future research should focus on external validation, impact on hard end points, and equitable deployment across real-world settings. This approach may help close the current diagnostic and therapeutic gaps in DKD management.

## 1 Background and disease burden

Diabetes has emerged as one of the most prevalent and fastest-growing chronic non-communicable diseases worldwide. As of 2024, an estimated 589 million adults aged 20 to 79 years—approximately one in every nine individuals—are living with diabetes, with around 3.4 million deaths attributed to diabetes each year (1). For the first time, global direct healthcare expenditures on diabetes have surpassed USD 1 trillion (2). By 2050, the number of individuals affected in this age group is projected to rise to 853 million. Concurrently, estimates from the Global Burden of Disease study suggest that more than 1.3 billion people of all ages will be living with diabetes by 2050, highlighting an ongoing global escalation in disease prevalence (3).

A substantial portion of diabetes-related mortality and economic burden is driven by its complications, including cardiovascular disease, kidney disease, neuropathy, and retinopathy (4). Among these, diabetic kidney disease (DKD) is particularly insidious—often progressing without symptoms yet offering a potential therapeutic window during which timely, evidence-based interventions can significantly delay renal deterioration (5).

Improving clinicians’ awareness and understanding of chronic kidney disease, particularly DKD, is essential to ensure the prompt initiation of such interventions. Evidence-based therapies—such as SGLT2 inhibitors, non-steroidal MRAs, and GLP-1 receptor agonists—can decelerate renal function decline, mitigate metabolic complications, and reduce cardiovascular risk (6–8). Prioritizing early detection and personalized management of DKD, therefore, constitutes a strategic opportunity for global public health efforts.

### 1.1 Rationale and use cases

Early risk stratification for DKD should prioritize adults with type 2 diabetes in primary care, endocrinology, and nephrology settings, including those with normal or mildly reduced kidney function (G1–G3a) and normo- to micro-albuminuria. Priority subgroups include long diabetes duration, hypertension or ASCVD, history of preeclampsia, rapid estimated glomerular filtration rate(eGFR) decline, family history of CKD, and marked glycemic or blood-pressure variability on continuous glucose monitoring (CGM)/ambulatory blood pressure monitoring (ABPM). The window of intervention is pre-albuminuria through early micro-albuminuria. Screening hinges on annual eGFR plus uACR (earlier/more frequent in high-risk profiles), followed by confirmation with repeat uACR (two of three positives over ~3 months), exclusion of transient causes (UTI, fever, strenuous exercise, menstruation), and standardized first-morning sampling.

**Figure 1:**
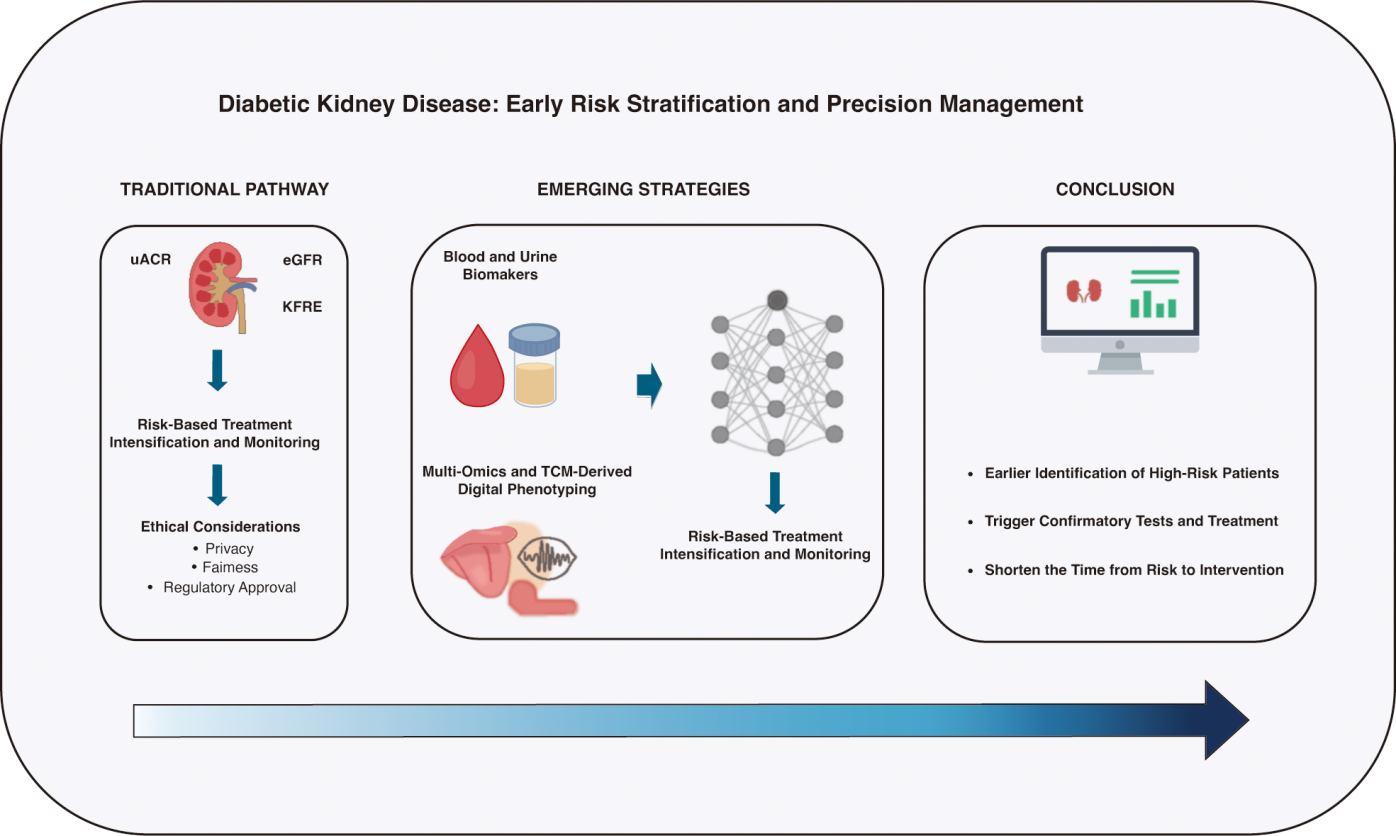
From guideline-based screening to multimodal, risk-driven management in diabetic kidney disease. The left panel illustrates the current KDIGO 2024–aligned screening and staging pathway for patients with type 2 diabetes: annual or risk-based measurement of eGFR and uACR, confirmation of abnormal uACR on repeat testing, and risk categorization using the eGFR×uACR heat map and KFRE. The right panel depicts how emerging multimodal technologies can be layered on top of this foundation to refine early risk stratification and guide treatment intensification. Molecular and fluid biomarkers (e.g., TNFR1/2, KIM-1, CKD273, urinary extracellular vesicles), tissue-level imaging metrics (multiparametric renal MRI, shear wave elastography), and digital phenotypes (tongue and pulse features, CGM and ABPM-derived metrics) feed into longitudinal machine learning models that estimate short- and medium-term risk of kidney failure and cardio–renal events. These estimates, in turn, inform stepped intensification of RAAS blockade, SGLT2 inhibitors, non-steroidal MRAs, and GLP-1 receptor agonists, as well as follow-up frequency and nephrology referral. eGFR, estimated glomerular filtration rate; uACR, urine albumin-to-creatinine ratio; KFRE, Kidney Failure Risk Equation; CGM, continuous glucose monitoring; ABPM, ambulatory blood pressure monitoring; TCM, Traditional Chinese Medicine; MRI, magnetic resonance imaging; SWE, shear wave elastography; RAAS, renin–angiotensin–aldosterone system; MRA, mineralocorticoid receptor antagonist; GLP-1, glucagon-like peptide-1.

Actions are then mapped to clinical triggers. Risk stratification combines the KDIGO eGFR×uACR heat map, eGFR slope, and—where available—risk equations (e.g., KFRE), with optional augmenters such as urine proteomic panels, MRI/SWE, and genetic/epigenetic scores; longitudinal CGM/ABPM metrics and research-grade tongue/pulse features can be layered for hypothesis generation. Treatment follows a stepwise pathway: RAAS blockade and SGLT2 inhibitors as foundations (if eligible), intensification with non-steroidal MRAs for persistent albuminuria, consideration of GLP-1RAs for weight and cardiovascular risk, and systematic optimization of BP, glycemia, lipids, and adherence/lifestyle. Monitoring cadence scales with risk (e.g., 12/6/3 months), tracking uACR trend, eGFR slope, BP/CGM metrics, and safety labs (serum potassium, eGFR), with re-stratification after therapy changes or acute events. This section anchors the subsequent framework that operationalizes these steps into deployable workflows. The decision nodes and corresponding actions are summarized in a pragmatic table for deployment in routine care (**Table 1**). To provide an overview, **Figure 1** illustrates how traditional markers (eGFR, uACR, KFRE) are being complemented by emerging multimodal strategies—including omics, TCM-derived digital phenotyping, and machine-learning–based longitudinal modeling—to achieve earlier and more personalized intervention.

**Table 1:** Trigger-to-action map for early DKD risk stratification and precision management.

Step	Clinical triggers (examples)	Required data	Decision rule (examples)	Recommended actions	Re-check interval*
Screen	Adults with T2D who have high-risk profiles, such as long diabetes duration, hypertension or ASCVD, prior preeclampsia, a family history of CKD, rapid eGFR decline, or marked CGM/ABPM variability.	Annual measurement of eGFR and uACR. Test earlier and more often in high-risk patients.	Screen is positive if uACR is ≥30 mg/g or if there is a rapid eGFR decline, for example more than 5 mL/min/1.73 m² per year or a reduction of at least 25% from baseline.	Arrange confirmatory testing. Begin lifestyle, blood-pressure, and glycaemic optimisation.	Low-risk: repeat testing every 12 months. High-risk: repeat every 6 months.
Confirm	Any abnormal screening result.	Repeat uACR (two of three measurements over about three months). Exclude transient causes such as urinary tract infection, fever, strenuous exercise, or menstruation. Use a first-morning urine sample when possible.	Albuminuria is confirmed if the elevation persists on repeat testing.	Stage CKD using KDIGO categories. Obtain baseline laboratory tests (potassium, HbA1c, lipids) and perform renal ultrasound when clinically indicated.	Repeat uACR after about three months to confirm. Afterwards, follow the interval recommended for the patient’s risk category.
Stratify	After confirmation of abnormal results or in patients with unexplained decline in kidney function.	Current eGFR, uACR, and eGFR slope. Calculate a risk equation such as the KFRE. When available, consider additional information from urine proteomics, renal MRI or shear wave elastography, polygenic or epigenetic markers, and longitudinal CGM or ABPM data.	Assign risk using the KDIGO heat map and apply local KFRE thresholds for referral or escalation of treatment.	Label the patient’s risk stratum. Set an appropriate follow-up schedule. Use additional tests only when the results are likely to change management.	According to the recommended interval for each risk stratum (for example as summarized in **Table 2**).
Treat	Any treatable risk factor or persistent albuminuria.	Eligibility for RAAS blockade, SGLT2 inhibitors, and non-steroidal MRAs. Blood pressure, HbA1c, and lipid profile. History of treatment adherence and previous adverse events.	If albuminuria persists despite maximally tolerated RAAS blockade and SGLT2 inhibition, consider adding a non-steroidal MRA. Consider a GLP-1 receptor agonist in patients with obesity or high cardiovascular risk.	Start or optimise RAAS blockade and SGLT2 inhibitors when the patient is eligible. Add a non-steroidal MRA in patients with persistent A2 or A3 albuminuria. Consider GLP-1 receptor agonists. Provide structured lifestyle counselling and adherence support.	Check safety laboratories such as potassium and eGFR 4–12 weeks after changes in therapy.
Monitor	All risk strata.	Trends in uACR, eGFR slope, blood pressure, and CGM metrics, plus safety laboratories such as potassium and eGFR.	Escalate treatment or refer if albuminuria or eGFR slope worsens or if KFRE risk crosses predefined thresholds.	Re-stratify risk after changes in therapy or acute events and adjust the follow-up schedule accordingly.	Low risk: every 12 months. Moderate risk: every 6 months. High risk: every 3 months.

*Intervals are example cadences; align final thresholds and timing with local policy and guidelines. T2D, type 2 diabetes; HTN, hypertension; ASCVD, atherosclerotic cardiovascular disease; CKD, chronic kidney disease; eGFR, estimated glomerular filtration rate; uACR, urine albumin-to-creatinine ratio; KFRE, Kidney Failure Risk Equation; PRS, polygenic risk score; ns-MRA, non-steroidal mineralocorticoid receptor antagonist; SWE, shear wave elastography; BP, blood pressure; CGM, continuous glucose monitoring; ABPM, ambulatory blood pressure monitoring; AE, adverse event.

### 1.2 Literature search and selection

This narrative review was informed by a structured but non-exhaustive literature search focusing on emerging tools for early risk stratification and precision management of DKD. We conducted electronic searches in PubMed/MEDLINE, Embase, and Web of Science for English-language articles published between January 2014 and October 2025.

In PubMed, an initial broad query combined DKD terms with biomarker and digital-health concepts, for example:”diabetic kidney disease” OR “diabetic nephropathy” AND (“biomarker*” OR “proteomic*” OR “metabolomic*” OR “genetic*” OR “epigenetic*” OR “urinary extracellular vesicles” OR “imaging” OR “MRI” OR “shear wave elastography” OR “digital biomarker*” OR “mobile health” OR “wearable*” OR “machine learning” OR “artificial intelligence” OR “tongue diagnosis” OR “pulse wave” OR “Traditional Chinese Medicine”).This broad search yielded a large number of citations. We therefore further refined the strategy by restricting terms to title/abstract fields and by adding risk/prognostic concepts (e.g., risk, prediction, prognos, risk score, risk model, risk stratification, progression), and we applied filters for humans and English-language publications.

We prioritized original human studies and high-quality systematic reviews that evaluated prediction, early detection, or monitoring of DKD progression using clinically relevant endpoints such as eGFR slope, sustained ≥40% eGFR decline, incident kidney failure, or cardio–renal composite outcomes. Experimental work in cell and animal models, and purely methodological or technical papers without direct clinical application, were included selectively when they clarified mechanisms or key limitations of the technologies. Reference lists of key articles and recent guidelines (e.g., KDIGO 2024 CKD guideline) were screened to identify additional relevant publications. Given the heterogeneity of study designs and outcomes, we did not perform a formal meta-analysis; instead, we synthesized the evidence qualitatively, emphasizing study design, sample size, validation status, and readiness for clinical translation.

## 2 Rationale for prioritizing DKD in early diagnosis and intervention

DKD represents a strategically valuable entry point for early diagnosis and intervention among diabetes-related complications, owing to its considerable public health burden and modifiable disease trajectory (9). Diabetes is now the leading global cause of chronic kidney disease (CKD) and end-stage kidney disease (ESKD), and constitutes the most common indication for dialysis and kidney transplantation in many countries (10). Early upstream detection and interruption of DKD progression, therefore, have the potential to substantially reduce the societal burden of renal replacement therapy, premature mortality, and long-term disability (9).

In its initial stages, DKD typically progresses silently and without symptoms; however, a therapeutic window still exists during which the disease course can be delayed or even partially reversed through timely, evidence-based intervention (11). Recent clinical trial data have demonstrated that early and sufficiently intensive treatment in high-risk individuals can favorably alter long-term outcomes (6, 12). Notably, the FLOW trial confirmed that semaglutide, a GLP-1 receptor agonist, significantly reduced the incidence of composite kidney outcomes and cardiovascular mortality in patients with type 2 diabetes and CKD, thus reinforcing the value of early detection and intervention (12).

From an implementation standpoint, DKD lends itself to scalable and standardized risk stratification due to its streamlined, guideline-endorsed screening framework. The KDIGO 2024 guidelines recommend that all individuals with high-risk or suspected diabetes undergo simultaneous measurement of eGFR and uACR, with repeat testing advised to exclude acute fluctuations (5). Where resources permit, combined creatinine–cystatin C estimation (eGFRcr-cys) is encouraged to improve CKD staging precision and guide therapeutic decisions.

Subsequent risk categorization is based on an eGFR–uACR heatmap, complemented by validated risk equations such as the Kidney Failure Risk Equation (KFRE) to quantify absolute prognosis (13). For example, in CKD stages G3–G5, a 5-year risk ≥3–5% warrants nephrology referral, while a 2-year risk >10% indicates the need for multidisciplinary management. This “screen–confirm–stratify–refer/follow-up” loop provides a unified clinical framework that accommodates the integration of novel biomarkers, imaging metrics, and digital phenotypes on top of existing standard-of-care pathways (14).

Importantly, when high-risk stratification is directly linked to disease-modifying therapy, it evolves from a descriptive label to an actionable decision node. Within the same calibration and reclassification architecture, clinicians may layer additional modalities—such as imaging features, circulating biomarkers, and wearable-derived metrics—to refine individual risk prediction and provide early warnings during longitudinal follow-up (15–17). Among these, tongue and pulse characteristics rooted in Traditional Chinese Medicine (TCM) diagnostic paradigms are being translated into quantifiable digital phenotypes. These may serve as weak labels or correction terms in composite risk models (18, 19). Specific methodologies and supporting evidence are discussed in detail in subsequent sections.

## 3 Emerging technologies for early risk stratification of DKD

To extend the detection window of diabetic kidney disease (DKD), it is essential to enhance the current KDIGO risk stratification framework—based on eGFR, uACR, and the Kidney Failure Risk Equation (KFRE)—through the systematic integration of molecular, tissue, and continuous exposure-level data. These emerging approaches must be evaluated against externally validated hard clinical endpoints, such as annual eGFR slope, sustained ≥40% eGFR decline, progression to end-stage kidney disease (ESKD), or composite kidney–cardiovascular outcomes, to determine their true predictive and clinical utility (20, 21). This section summarizes key technologies, application scenarios, and methodological considerations across a “molecular–tissue–exposure–algorithm” continuum.

### 3.1 Genetics and epigenetics

Large-scale biobanks and multi-ethnic meta-analyses have significantly accelerated the identification of susceptibility loci and mechanistic insights related to DKD (22, 23). Recent studies in type 1 diabetes (T1D) cohorts have highlighted robust loci such as protective variants in COL4A3 (e.g., *rs55703767*), as well as signals in AFF3, FRMD3, and the RGMA–MCTP2 region, which are consistently associated with albuminuria, eGFR decline, and ESKD (24, 25). Multi-ancestry initiatives such as the FIND study demonstrate directional consistency across diverse populations, a critical feature for translational generalizability (25).

By contrast, the greater phenotypic heterogeneity in type 2 diabetes (T2D)—due to comorbidities such as hypertension, obesity, and dyslipidemia—has limited the detection of genome-wide significant loci (26). This underscores the need for refined phenotyping and larger, multi-ethnic cohorts to improve signal stability. Functional annotation strategies that integrate GWAS signals with kidney-specific eQTL, TWAS, and cell-type-resolved transcriptomic data are increasingly bridging the gap between statistical association and mechanistic interpretation.

#### 3.1.1 Polygenic risk scores (PRS)

A 2024 systematic review suggests that polygenic risk scores (PRS) derived from existing GWAS datasets can identify individuals at elevated risk for DKD at the population level (27). However, predictive performance varies with ancestry, phenotype definition, and baseline risk distributions. In real-world settings—particularly those involving mixed ancestry, comorbid cardiovascular disease, or variable medication exposure—model calibration may drift, reducing clinical reliability (28).

Best practices for PRS development and deployment include multi-ancestry training, independent external validation, pre-registered thresholds, and transparent clinical interpretability (27). In this context, PRS may be best utilized not for population-wide screening or as replacements for KDIGO models, but rather as tools for mechanistic stratification or enrichment of clinical trial populations—especially for identifying genetically high-risk individuals with borderline or subclinical phenotypes.

#### 3.1.2 Epigenetic reprogramming and single cell multi-omics

Epigenetic modifications associated with “metabolic memory”—including DNA methylation and histone remodeling—can persist despite glycemic normalization and contribute to podocyte injury, interstitial fibrosis, oxidative stress, and inflammation (29). Recent reviews (2024–2025) have mapped key epigenetic alterations in renal parenchymal cells, including dynamic shifts in DNA methylation and H3K4/H3K27 histone marks (30, 31). Although pharmacologic targeting of epigenetic pathways is promising, robust human data linking these features to hard renal outcomes remain scarce (32).

In parallel, single-cell and spatial multi-omics technologies are rapidly advancing nephrology research (33, 34). Platforms such as scRNA-seq and scATAC-seq allow for precise spatiotemporal mapping of cell-type-specific processes, linking genetic predisposition and epigenetic reprogramming to molecular phenotypes and microenvironmental signals (35).

In the near term, a practical application may lie in multimodal predictive modeling that incorporates epigenetic and transcriptomic features alongside clinical variables, imaging, and fluid biomarkers to refine early risk stratification and subtype identification (34). However, these models require validation in multi-center prospective cohorts with hard endpoints (e.g., eGFR slope, ESKD, cardiorenal events) to establish their incremental predictive value.

#### 3.1.3 Integrative perspective

Current evidence supports an interactive model in which genetic predisposition, environmental exposures, and epigenetic reprogramming converge to shape DKD trajectory (27, 36). PRS may serve as tools for mechanistic stratification or high-risk trial recruitment, while epigenetic and single-cell approaches guide pathway-based intervention and target prioritization. Successful clinical translation will depend on external multi-ancestry validation, alignment with human kidney tissue data, and demonstration of added predictive utility anchored to meaningful clinical outcomes.

### 3.2 Optimization and standardization of core laboratory markers

eGFR and uACR remain the cornerstone biomarkers for early detection and longitudinal monitoring of DKD (37). However, their clinical utility depends on strict standardization of measurement and interpretation to ensure accuracy and consistency across diverse healthcare settings.

According to KDIGO 2024, serum creatinine–based eGFR (eGFRcr) is recommended for initial screening, while combined creatinine–cystatin C equations (eGFRcr-cys) should be prioritized in decision-critical scenarios when available (5, 38). All estimation equations should exclude race-based coefficients (39). For uACR, abnormal values must be confirmed on repeat testing, and risk stratification should be based on the joint interpretation of eGFR and uACR.

#### 3.2.1 Systematic bias in creatinine measurement

Despite global efforts in assay standardization, serum creatinine remains susceptible to both methodological and interference-related variability. Discrepancies between enzymatic and Jaffe methods, as well as inter-laboratory variation, can result in inconsistent eGFR values and CKD staging errors. External quality assessments and multi-center evaluations continue to detect residual measurement differences even under standardized protocols (40).

Common analytical interferences include falsely low enzymatic readings due to dopamine or dobutamine administration, pseudo-hypocreatininemia caused by IgM paraproteinemia, and interference from N-acetylcysteine. These factors are especially relevant when eGFR values approach clinical decision thresholds (e.g., for drug dosing or nephrology referral) and warrant confirmatory testing or the use of alternative biomarkers when appropriate (41, 42).

#### 3.2.2 Improved estimation equations

The 2021 CKD-EPI equations, which exclude race and incorporate cystatin C, offer improved accuracy and reduced demographic bias compared to traditional creatinine-only models (39). In 2023, a further refinement was proposed: a cystatin C–C-based formula that also excludes sex, offering a robust alternative in patients with abnormal muscle mass, atypical creatinine kinetics, or near dosing thresholds (43).In clinical practice, eGFRcr-cys or cystatin C–only equations should be considered at decision points where misclassification may have therapeutic consequences, provided testing infrastructure allows (14, 38).

#### 3.2.3 Measured GFR as the analytical gold standard

When eGFR estimates are discordant with clinical presentation, or in cases involving extreme body composition, abnormal creatinine/cystatin C kinetics, or critical research/drug-dosing scenarios, direct measurement of GFR (mGFR) is warranted. Standardized plasma clearance techniques using non-radioactive tracers (e.g., iohexol) have demonstrated feasibility and reproducibility in both clinical and research settings, and serve as the gold standard for GFR assessment (44).

#### 3.2.4 Standardization of uACR measurement

Like eGFR, uACR measurement requires rigorous standardization (45). Reference methods such as LC-MS/MS and external proficiency testing have revealed systematic discrepancies between routine immunoassays and isotope dilution mass spectrometry (IDMS) (46). Reference materials—such as NIST SRM 2925 (recombinant human albumin)—enable traceability and help reduce inter-laboratory variability, thereby improving comparability in longitudinal studies and multicenter trials (47). Clinical laboratories should disclose the traceability and uncertainty of their assays. In research contexts, preference should be given to externally validated and calibrated platforms.

#### 3.2.5 Analytical calibration as a prerequisite for biomarker integration

Robust analytical calibration at the foundational biomarker level is a prerequisite for the meaningful integration of novel molecular, imaging, or digital phenotyping tools. Without such calibration, perceived gains in prediction may reflect the correction of baseline measurement error rather than the true biological signal. Therefore, technical rigor in eGFR and uACR assessment is essential to support the valid clinical deployment of emerging diagnostic technologies.

### 3.3 Inflammatory and tubular injury biomarkers

Beyond eGFR and uACR, circulating biomarkers reflecting inflammation and tubular injury have shown consistent and reproducible associations with DKD progression (48, 49). Among these, soluble tumor necrosis factor receptors 1 and 2 (TNFR1/2) and kidney injury molecule-1 (KIM-1) represent the most robust biomarker axis with established clinical relevance (50, 51).

Prospective cohort studies across type 1 and type 2 diabetes populations have demonstrated that elevated TNFR1/2 levels—whether at baseline or over time—are independently associated with accelerated eGFR decline, progression to CKD stage 3 or higher, and increased risk of end-stage kidney disease (ESKD), independent of albuminuria and traditional risk factors (50, 52, 53). KIM-1, a marker of proximal tubular damage, has also been consistently linked to adverse renal outcomes in various cohorts (54, 55).

These findings suggest that inflammatory and tubular injury biomarkers can provide additive prognostic information for intermediate- and long-term renal outcomes, beyond standard KDIGO risk stratification based on eGFR and uACR (49, 56).

#### 3.3.1 Clinical integration: The KidneyIntelX.dkd model

KidneyIntelX.dkd is a multi-biomarker machine learning–based risk score that integrates TNFR1, TNFR2, and KIM-1 with key clinical variables such as eGFR, uACR, HbA1c, and blood urea nitrogen (56, 57). This model has received *De Novo* authorization from the U.S. Food and Drug Administration (FDA) for predicting the 5-year risk of progressive kidney function decline in adults with diabetes and early-stage DKD.

The tool supports individualized care pathways by informing follow-up intensity, referral to nephrology, or clinical trial enrollment (58). Both derivation/validation studies and real-world implementations have shown that combining biomarkers with clinical parameters improves discrimination and facilitates actionable risk stratification (56, 58, 59).

#### 3.3.2 Population-specific considerations and therapeutic responsiveness

Biomarker performance may vary across diabetes subtypes and disease stages (48). For example, in type 1 diabetes, the independent predictive value of urinary KIM-1 for ESKD is attenuated after adjusting for albuminuria, indicating its potential utility in early-stage disease or in conjunction with inflammatory markers (60).

While TNFR1/2 are more robust than many other inflammatory biomarkers, their levels may be affected by systemic inflammation, limiting disease specificity (61). Therefore, clinical interpretation should account for comorbid conditions and be contextualized within the broader phenotypic landscape.

Emerging evidence also suggests that these biomarkers are modifiable by pharmacologic interventions. Randomized trials of sodium–glucose cotransporter 2 (SGLT2) inhibitors have shown reductions in KIM-1 and modest decreases in TNFR1/2, indicating their potential utility for pharmacodynamic monitoring and treatment response assessment (62, 63).

#### 3.3.3 Methodological and implementation considerations

To enable clinical translation of these biomarkers, robust analytical validation, external performance verification, and seamless clinical integration are essential. Assay platforms should clearly define the sample matrix (e.g., serum or plasma), calibration protocols, precision parameters, and dynamic ranges to ensure inter-laboratory comparability (64). Predictive models must undergo external validation across ethnically and geographically diverse cohorts using standardized outcomes such as ≥40% sustained eGFR decline, ESKD, or composite renal/cardiovascular events (65). Reporting should include discrimination (C-statistic, AUC), calibration (slope, Brier score), and clinical utility metrics (e.g., NRI, IDI, decision curve analysis) (66). Integration into routine care should align with the KDIGO 2024 framework, with predefined trigger thresholds for nephrology referral and follow-up intensity. Post-implementation strategies must include recalibration procedures and monitoring for model performance drift (67).

#### 3.3.4 Summary and clinical implications

Inflammatory (TNFR1/2) and tubular injury (KIM-1) biomarkers offer independent prognostic value beyond traditional markers and can aid in early identification of high-risk patients, individualized care planning, and enrichment of clinical trial cohorts. However, widespread clinical adoption will require harmonization of assay platforms, validation across diverse populations, and the accumulation of real-world evidence demonstrating improvement in patient reclassification and clinical outcomes.

### 3.4 Urinary proteomics and the CKD273 classifier

Urinary proteomics has emerged as a promising modality for the early detection and risk stratification of DKD (68). Among the various platforms, the 273 urinary peptides (CKD273) classifier—developed using capillary electrophoresis coupled with mass spectrometry (CE-MS)—is currently the most extensively validated urinary peptide panel for kidney risk assessment (69).

In individuals with type 2 diabetes and normoalbuminuria at baseline, elevated CKD273 scores have been shown to predict progression to microalbuminuria within 2–3 years, independent of changes in uACR (70). This prognostic value has been confirmed in large-scale studies, including the PRIORITY trial and the DIRECT-Protect 2 sub-study, supporting its utility as a preclinical risk identifier that precedes overt albuminuria and facilitates early screening and enrichment in clinical trials (70, 71).

#### 3.4.1 Responsiveness to pharmacologic interventions

In a randomized, double-blind, crossover trial, the addition of dapagliflozin for 12 weeks on top of background renin–angiotensin system (RAS) inhibition significantly reduced CKD273 scores and shifted a subset of individuals from a high-risk to a low-risk peptide profile. These findings position CKD273 as a potential pharmacodynamic marker of treatment response (72). Similar proteomic modulation has been observed with empagliflozin, suggesting a class effect of SGLT2 inhibitors on the urinary peptide signature (73). Conversely, the addition of spironolactone in patients with high CKD273 scores failed to prevent microalbuminuria onset, indicating limited evidence for mineralocorticoid receptor antagonist (MRA) therapy guided by this biomarker at present (70). Overall, CKD273 appears more suited as an early “trigger” and a treatment response indicator, rather than a diagnostic surrogate (69).

#### 3.4.2 Methodological considerations for clinical translation

The development and validation of CKD273 have predominantly relied on CE-MS (74). Despite its strong intra- and inter-assay reproducibility, variability introduced by batch effects, platform heterogeneity (e.g., CE-MS vs. LC-MS/MS), and pre-analytical procedures (e.g., centrifugation, desalting, freeze–thaw cycles) remains a major challenge for multicenter deployment (75, 76). To ensure analytical reliability and comparability, strict quality control, batch normalization, and inter-laboratory calibration protocols must be explicitly implemented in both research and clinical settings.

#### 3.4.3 Suggested clinical applications and evaluation framework

The CKD273 classifier may serve as a complementary biomarker layer atop standard KDIGO-based risk stratification (e.g., eGFR × uACR, KFRE), with potential applications in three distinct clinical scenarios (69). First, in patients with borderline or fluctuating uACR levels, a positive CKD273 result could trigger enhanced surveillance strategies such as intensified uACR retesting or ambulatory blood pressure monitoring(ABPM). Second, in high-risk individuals without overt proteinuria, CKD273 can help identify subpopulations prone to rapid progression, thereby improving event rates and statistical power in clinical trials. Third, in therapeutic monitoring, a post-treatment reduction in CKD273 score may serve as a pharmacodynamic marker when interpreted alongside uACR decline and eGFR slope stabilization. To ensure clinical relevance, all studies evaluating CKD273 should report not only discrimination metrics (e.g., C-statistic, AUC), but also calibration (e.g., calibration slope, Brier score) and clinical utility indices (e.g., NRI, IDI, decision curve analysis). Furthermore, external validation should prioritize hard clinical endpoints such as sustained ≥40% eGFR decline, ESKD, or cardio–renal composite events to demonstrate incremental prognostic value beyond existing tools.

#### 3.4.4 Positioning and future directions

CKD273 offers incremental risk information before overt albuminuria and exhibits quantifiable responsiveness to SGLT2 inhibitor therapy. Its optimal role is as an early activation signal and a treatment monitoring tool layered on top of standard care. Future directions include large-scale, multi-center prospective studies with harmonized protocols, validation against hard clinical endpoints, and generation of real-world evidence to demonstrate reclassification benefit and net clinical impact.

### 3.5 Urinary extracellular vesicles

Urinary extracellular vesicles (uEVs) are emerging as a promising biomarker modality for DKD, offering several advantages for translational research (77). They originate from various nephron segments, are noninvasively and repeatedly collectible, and reflect dynamic renal pathophysiology. The cargo of uEVs—including proteins and microRNAs—provides insight into glomerular and tubular compartments, enabling their use as mechanistic surrogates, early risk indicators, and potential pharmacodynamic markers (78). Podocyte-derived proteins have been linked to albuminuria, while tubular injury markers such as aquaporins and transporters may reflect tubulointerstitial damage.

#### 3.5.1 Evidence and limitations of clinical application

Although uEVs show potential for early stratification and mechanistic mapping, there remains a lack of large-scale, prospective validation studies using hard renal endpoints such as sustained eGFR decline, ESKD, or cardio–renal composite outcomes (79). Current evidence is primarily derived from cross-sectional or small longitudinal cohorts (80). To assess incremental value, standardized phenotype definitions and methodological consistency are essential.

#### 3.5.2 Technical standardization and reporting guidelines

Technical challenges specific to uEVs include variability in sample dilution, low protein concentrations, and contamination from non-renal sources. Recent international guidelines emphasize the importance of unified nomenclature, standardized isolation techniques such as ultracentrifugation and immunocapture, and normalization approaches using urinary creatinine, volume, or vesicle-specific markers (80). Reporting standards should include particle count, protein content, and characterization markers, alongside inter-batch and cross-platform quality control.

#### 3.5.3 Short-term positioning: augmentation layer rather than standalone tool

At the current stage of evidence, uEVs are best regarded as a complementary biomarker layer rather than a replacement for conventional tools (80). When used in conjunction with urinary proteomics or serum markers, uEVs may enhance risk stratification within the KDIGO framework. Evaluation should include not only discrimination metrics but also calibration performance, reclassification indices, and clinical decision impact analyses, particularly concerning testing frequency, referral timing, and therapeutic escalation.

#### 3.5.4 Research priorities and marker candidates

Research should prioritize the development of multi-ethnic prospective cohorts, harmonized pre-analytical workflows, and validation strategies anchored to hard clinical endpoints (78). Candidate biomarkers of interest include podocyte-derived proteins and associated microRNAs, as well as vesicular cargo originating from distinct tubular segments or signaling pathways (81, 82). These may support the delineation of tubulointerstitial-dominant phenotypes and enable more personalized treatment approaches. Ultimately, uEVs hold potential as part of an integrated, multi-modal biomarker strategy for early detection, risk classification, and treatment monitoring in DKD.

### 3.6 Imaging-based characterization of renal tissue injury

#### 3.6.1 Overview of imaging modalities

Recent advances in non-invasive imaging technologies have enabled the direct visualization and quantification of renal tissue alterations that precede detectable changes in conventional biomarkers such as eGFR and uACR (83). Among these modalities, multiparametric magnetic resonance imaging (MRI) has emerged as a robust tool for assessing renal perfusion, microstructure, and oxygenation (84). Techniques such as arterial spin labeling (ASL) quantify cortical blood flow; diffusion-weighted and diffusion tensor imaging (DWI/DTI) evaluate tissue diffusivity and microstructural integrity; and blood oxygen level–dependent (BOLD) MRI captures tissue oxygenation through deoxyhemoglobin signal variation (85). Collectively, these metrics can detect early renal alterations in individuals with diabetes, even before a measurable decline in kidney function (86).

#### 3.6.2 Standardization and technical challenges

To facilitate clinical translation, initiatives such as PARENCHIMA have proposed standardized acquisition, processing, and reporting protocols across ASL, DWI, BOLD, and T1/T2 mapping (84). These guidelines emphasize the harmonization of pre-scan variables—hydration status, sodium intake, body positioning—and recommend prioritizing cortical regions to improve reproducibility across imaging platforms. Despite these advances, widespread adoption of renal MRI remains limited by high cost, extended scan durations, and inter-vendor variability (87).

#### 3.6.3 Emerging role of shear wave elastography

In addition to MRI, shear wave elastography (SWE) has been investigated as a non-invasive surrogate for renal fibrosis (88). By quantifying cortical stiffness, SWE offers insight into interstitial fibrosis, supported by biopsy-correlated studies and meta-analyses. Combining SWE with conventional biomarkers (e.g., eGFR) may enhance discrimination of fibrosis stages (88). However, SWE is highly operator- and device-dependent and lacks standardized thresholds across platforms, limiting its broad clinical utility.

#### 3.6.4 Suggested clinical applications

These imaging tools are best positioned as adjunctive modalities in selected clinical contexts rather than as general screening instruments. Their integration is particularly valuable in three scenarios (1): enriching clinical trial populations by identifying individuals with greater subclinical tissue injury—such as impaired perfusion, restricted diffusion, or increased cortical stiffness—thereby improving event rates and trial efficiency (2); monitoring therapeutic response in patients receiving renoprotective interventions (e.g., SGLT2 inhibitors, mineralocorticoid receptor antagonists, GLP-1 receptor agonists), where dynamic imaging changes may complement conventional markers like uACR and eGFR slope; and (3) evaluating the incremental prognostic value of imaging biomarkers in prospective cohorts anchored by hard clinical endpoints (e.g., sustained ≥40% eGFR decline, ESKD, or cardio–renal composite outcomes), with emphasis on reclassification performance and net clinical benefit (89).

#### 3.6.5 Future directions and research needs

Multiparametric renal MRI and shear wave elastography (SWE) offer valuable insights into subclinical tissue-level injury and hold promise as precision imaging biomarkers within emerging nephrology frameworks (90). Their optimal role lies in augmenting, rather than replacing, existing clinical assessments, particularly in early detection and treatment response monitoring. Moving forward, priorities include the development of harmonized acquisition protocols, robust multicenter validation across imaging platforms, and demonstration of additive prognostic value over conventional tools such as eGFR and uACR. Additionally, integration with clinical decision pathways will require health-economic evaluations, workflow optimization, and regulatory recognition to support scalable adoption.

### 3.7 Machine learning and longitudinal modeling for risk prediction in DKD

The progression of DKD is a time-dependent process, so models that use only a single baseline visit often miss patients who will deteriorate quickly (91). Using information over time—for example eGFR slope, change in uACR, and patterns of medication use—can make risk estimates more stable and more useful for clinical decisions. Continuous data from wearable devices such as CGM and ABPM also describe day-to-day metabolic and blood-pressure stress in much greater detail (92). When prediction models are trained to forecast hard outcomes, such as a sustained ≥40% eGFR decline, ESKD, or major cardio-renal events, they are directly aligned with thresholds that clinicians already recognize as actionable end points (93). Several studies show that eGFR slope is a useful surrogate for CKD progression and an important predictor in prognostic models, while CGM and ABPM provide additional phenotypic information for refining risk stratification (94, 95).

#### 3.7.1 Advantages of longitudinal modeling and data sources

Repeated measurements of eGFR and uACR allow the construction of individual trajectories rather than relying on a single value. Models that use these trajectories generally identify high-risk patients earlier and more accurately than static models based only on baseline measurements (96). Incorporating eGFR slope into prediction models has improved calibration for estimating 2-year risk of ESKD. Joint modeling approaches, which analyze biomarker trajectories and time-to-event outcomes together, further strengthen prediction and reduce bias when progression is variable or follow-up is incomplete (97, 98). In parallel, wearable-derived metrics such as CGM-based glycaemic variability and ABPM-based blood-pressure fluctuations provide high-resolution information on exposure to harmful metabolic and hemodynamic stress, and these metrics correlate with adverse cardio-renal outcomes. For these reasons, longitudinal and high-frequency data streams are increasingly viewed as core components of modern DKD risk models (99, 100).

#### 3.7.2 Modeling paradigms and temporal methods

Several time-aware modeling strategies have been applied to DKD. Landmark or dynamic prediction frameworks update an individual’s risk estimate at predefined time points based on all information accumulated so far, which mirrors routine follow-up visits in outpatient care (101, 102). Time-dependent Cox models and joint models combine evolving biomarker values with survival outcomes, allowing risk to change as kidney function or albuminuria changes and reducing bias from informative dropout or censoring (103). Deep learning methods, including recurrent neural networks and architectures such as RETAIN and BEHRT, can integrate information from many time points and modalities while retaining some level of interpretability for clinicians (104). Regardless of the specific technique, it is essential to define the observation window (what history is used) and the prediction horizon (what time frame is being predicted), and to train and test models using hard clinical end points to avoid data leakage and to keep the results clinically meaningful (105).

#### 3.7.3 Multimodal fusion and missing data handling

In practice, DKD risk models can combine multiple data types—clinical variables, laboratory tests, omics, imaging markers and digital phenotypes. These can be fused at different stages of the modeling pipeline, such as early fusion (simple concatenation of features) or later fusion of intermediate representations. Real-world datasets often contain irregular sampling and missing values, which require appropriate handling through modality-specific masking, imputation strategies or dropout-based methods. Differences between hospitals, laboratories and devices can also degrade performance when a model is moved to a new setting. Domain-adaptation and federated-learning approaches have been proposed to reduce this problem and to allow multi-center model development without sharing raw patient data (106–108). A notable example of successful multimodal fusion is KidneyIntelX.dkd, which combines TNFR1, TNFR2, KIM-1 and routine clinical variables and has received FDA *De Novo* authorization for assessing DKD progression risk, illustrating that such pipelines can reach clinical-grade implementation.

#### 3.7.4 Validation, reporting, and fairness

Before deployment, DKD risk models require careful validation. This includes internal validation using approaches such as nested or time-split cross-validation, and external validation in independent cohorts that differ by center, device and patient mix. Key performance metrics include discrimination (AUROC, C-index), calibration (calibration slope, Brier score) and measures of clinical usefulness such as net reclassification improvement, integrated discrimination improvement and decision-curve analysis, ideally reported for different prediction horizons using time-dependent AUROCs (106). Model development and reporting should follow established guidelines such as TRIPOD-AI and PROBAST-AI, and should include fairness assessments across sex, age, ethnic and socioeconomic subgroups. Fairness metrics—for example equalized odds, calibration within subgroups and comparison of error rates—help to ensure that model performance is equitable across diverse populations (109).

#### 3.7.5 Post-deployment monitoring and recalibration

After a model is introduced into clinical practice, its performance can change as populations, treatments and measurement practices evolve. Therefore, ongoing monitoring is required to detect data drift, loss of calibration and deterioration in specific subgroups. Monitoring can use simple tools such as stability indices, calibration plots and periodic assessment of net clinical benefit. When pre-specified thresholds are crossed, a stepwise recalibration plan—starting with simple updates to the intercept and slope and escalating to partial or full retraining—should be implemented (110). Governance frameworks, including the FDA’s Good Machine Learning Practice (GMLP) and the NIST AI Risk Management Framework (AI RMF), provide guidance on documentation, audit trails and change-control processes that support safe model updating and lifecycle management (111). Repeated fairness audits and real-world outcome evaluations are also important to maintain trustworthiness over time (112).

#### 3.7.6 Representative applications and case studies

In multicenter CKD cohorts, joint models that incorporate eGFR slope together with time-to-event data have improved both calibration and discrimination for predicting kidney failure compared with simpler approaches (91). KDIGO guidelines support the use of ABPM for CKD risk stratification, and CGM is increasingly used in patients with advanced CKD and dialysis for time-in-range–based glycaemic management, providing rich inputs for risk models. KidneyIntelX.dkd is an example of a regulatory-grade model that combines biomarker and clinical data and has been evaluated in prospective and real-world settings, offering a practical template for future translational efforts (113). These examples show that clinically relevant, externally validated and equity-aware modeling frameworks for DKD risk stratification are feasible in real-world practice.

Overall, machine-learning models for DKD risk prediction should be built on longitudinal data, integrate multiple complementary data sources and be trained on clearly defined, clinically meaningful endpoints. Only models that undergo rigorous validation, fairness assessment and ongoing performance monitoring are likely to deliver sustained benefit in everyday care. Aligning these efforts with clinical guidelines such as KDIGO and KFRE, and with regulatory frameworks such as GMLP and AI RMF, is crucial for technical robustness, clinician confidence and wider adoption across diverse healthcare systems (114).

### 3.8 Integrating TCM-informed digital phenotypes into DKD risk stratification

#### 3.8.1 Conceptual rationale and digital modalities

Traditional Chinese Medicine (TCM) emphasizes *zheng* (syndrome) as a reflection of dynamic physiological imbalance (115). In the context of DKD, *zheng* can be reframed as a quantifiable digital phenotype that complements modern risk stratification (116). Advances in sensor and imaging technologies enable the digitization of classical TCM diagnostics: tongue imaging (inspection), pulse waveform acquisition (palpation), structured symptom questionnaires (inquiry), and potentially voice or breath sensing (listening/smelling) (117). Among these, tongue imaging and pulse signals are the most developed. Standardized tongue images yield features such as coating thickness, texture, and color, associated with inflammation, microcirculation, and metabolic status (118). Pulse waveforms captured via photoplethysmography (PPG) offer non-invasive measures of vascular compliance and autonomic tone (119).

#### 3.8.2 Technical standardization and multi-center reproducibility

To ensure multi-site generalizability, strict acquisition protocols are essential. For tongue imaging, a consistent light source, shooting angle, and exclusion of blurry or overexposed images are required (120, 121). Pulse signals must be acquired in quiet, temperature-controlled environments, with signal quality assurance. Device heterogeneity and environmental variability necessitate pre-specified normalization, calibration pipelines, and population-specific validation. These steps are crucial to establishing reliability before integration into predictive models or clinical workflows (122).

#### 3.8.3 Role in early risk detection and longitudinal monitoring

Digital phenotypes can enhance early DKD risk identification by detecting subclinical changes preceding proteinuria or eGFR decline (123). Their low cost, self-collectibility, and suitability for high-frequency monitoring make them valuable for continuous risk surveillance. When used alongside CGM, ABPM, laboratory indices, and imaging markers, these features may enrich predictive accuracy and personalize follow-up intensity. They also provide actionable signals in remote or resource-limited settings, enabling early alerts and behavioral interventions.

#### 3.8.4 Modeling strategy and real-world applications

TCM-derived digital features can be incorporated into multimodal machine learning models targeting hard renal endpoints such as sustained eGFR decline or dialysis initiation (123, 124). Studies using tongue image datasets have shown predictive utility for metabolic traits, while pulse waveform analyses correlate with arterial stiffness—a known predictor of renal outcomes (125, 126). These signals should be evaluated not only by AUROC but also by calibration, reclassification, and clinical decision utility. Promising use cases include screening, remote monitoring, and adjunctive response evaluation. Their deployment must ensure external validation, interpretability, and fairness across subpopulations.

#### 3.8.5 Limitations and comparison with established digital tools

While TCM-derived digital phenotypes offer an attractive, low-cost avenue for continuous monitoring, their current evidence base is substantially weaker than that of established digital health tools such as pulse wave analysis, ABPM, and continuous glucose monitoring (CGM). Most tongue and pulse studies are single-center, cross-sectional, or small longitudinal cohorts with heterogeneous inclusion criteria and limited adjustment for confounders. Acquisition protocols vary widely across devices and environments, which can introduce substantial measurement noise and reduce cross-site reproducibility.

Moreover, cross-cultural generalizability remains a key challenge. Tongue color, coating, and pulse characteristics can be affected by diet, oral hygiene, skin tone, and comorbid conditions, all of which may differ across populations. In contrast, CGM and ABPM have undergone large-scale, randomized and observational validation, are incorporated into international guidelines, and have well-defined clinical thresholds. At present, therefore, TCM-derived digital phenotypes should be regarded as exploratory signals that may enrich multimodal models or support remote hypothesis generation, but they cannot replace validated digital tools for risk stratification or treatment guidance.

#### 3.8.6 Ethical, fairness, and implementation considerations

Factors such as skin tone, oral hygiene, comorbidities, and acquisition environment can introduce bias. Models must report performance stratified by age, sex, ethnicity, and device type. Fairness-aware modeling strategies (e.g., reweighting, subgroup-specific training) are critical to mitigate disparities (120). Data privacy, auditability, and version control should be embedded through localized or federated learning pipelines. Ultimately, the value of these digital phenotypes will be determined by prospective validation, cost-effectiveness, and their ability to accelerate time-to-intervention within DKD care pathways.

### 3.9 Comparative appraisal of emerging technologies

To move beyond descriptive listing of individual modalities, **Table 2** provides a comparative overview of key emerging technologies for early DKD risk stratification. For each modality, we summarize typical study designs, approximate sample sizes, validation status, and current readiness for clinical translation. Overall, urinary proteomics (e.g., CKD273) and inflammatory/tubular biomarkers integrated into clinical-grade models such as KidneyIntelX.dkd are supported by the most consistent prospective data, including external validation and some regulatory authorization. By contrast, urinary extracellular vesicles, multimodal renal MRI, shear wave elastography, and TCM-derived digital phenotypes remain at an earlier stage, with promising mechanistic insights but limited large-scale, endpoint-anchored validation and scarce health-economic evaluation.

**Table 2:** Emerging technologies for DKD risk stratification: study characteristics, validation status, and clinical readiness.

Modality/exemplar	Representative clinical data (with sources)	Strength of evidence & key findings	Main strengths	Key limitations	Current clinical readiness
Inflammatory and tubular injury biomarkers (TNFR1, TNFR2, KIM-1; multi-biomarker risk scores such as KidneyIntelX.dkd)	Circulating TNFR1 and TNFR2 have been evaluated in multiple prospective cohorts of T1D and T2D. Studies in American Indians and other populations(n≈300–1,000)showed that higher TNFR1/2 levels predict incident stage 3 CKD and ESKD independent of traditional risk factors (50–52, 61). Plasma KIM-1 has been associated with early progressive renal decline in T1D, loss of kidney function and risk of ESKD in T2D and CKD cohorts(n≈300–3,000) (49, 54, 55, 60, 64). A multi-biomarker ML risk score(KidneyIntelX, later KidneyIntelX.dkd)was derived and validated in biobanks of patients with T2D and stage 1–3 CKD(derivation n=686, validation n=460) (56, 57), and subsequently evaluated in independent validation and real-world cohorts(>8,000 patients)with median follow-up up to 5–6 years (58, 59).	TNFR1/2 and KIM-1 consistently associate with faster eGFR decline, incident stage 3 CKD and ESKD across several cohorts (50–54,^49^, ^53^–^55^, ^60^, ^64^). Adding these markers to clinical models improves prediction of DKD progression and kidney failure. The KidneyIntelX.dkd test has undergone analytical and clinical validation and shows improved risk stratification for a composite endpoint of ≥40% eGFR decline or kidney failure in early DKD (56–59, 62, 63).	Strong and reproducible association with renal outcomes; incremental prognostic value beyond eGFR and uACR; blood-based assays compatible with high-throughput platforms; exemplified by a clinically implemented and regulated test(KidneyIntelX.dkd).	Inflammatory markers such as TNFR1/2 are not specific to DKD; assay harmonisation and cost may limit widespread implementation; most cohorts were in high-income countries; relatively few impact and cost-effectiveness studies.	High – multi-biomarker panels and ML scores can be used in selected patients with T2D and early CKD as adjuncts to KDIGO-based risk stratification and to inform follow-up intensity and nephrology referral (5, 14, 38, 59).
Urinary proteomics (CKD273 classifier)	The CKD273 urinary peptide classifier was developed from large CKD datasets and validated in multiple cohorts (69, 74). In the PRIORITY study, ≈2,000 normoalbuminuric adults with T2D from 15 centres in 10 European countries were tested with CKD273 and followed for incident persistent microalbuminuria and eGFR decline in an observational cohort and embedded randomized trial (70). Sub-studies from DIRECT and other prospective cohorts confirmed that CKD273 predicts onset of microalbuminuria and renal function loss in normoalbuminuric T2D (69, 71). Randomized trials have shown that CKD273 is responsive to SGLT2i and mineralocorticoid blockade (72, 73). Reproducibility of CE-MS–based urinary peptide detection has been demonstrated in external quality-assessment settings (68, 75).	Across European multicentre cohorts, a high-risk CKD273 score predicts incident microalbuminuria, accelerated GFR loss and mortality, and adds prognostic information beyond traditional risk markers (69–71, 74). CKD273 also behaves as a pharmacodynamic biomarker, improving with RAAS blockade and SGLT2 inhibition (72, 73). Analytical robustness of the CE-MS platform has been documented (68, 75).	Detects high-risk individuals before overt albuminuria; mechanistically rich multi-peptide signature; evaluated in large multicentre trials with standardized protocols; potential dual role as prognostic and treatment-response marker.	Requires specialized CE-MS technology; cost and limited platform availability; most data from European cohorts; health-economic and implementation studies are still limited; performance in other ethnic and health-system contexts is less well characterized (68, 69, 76).	Moderate – useful for early enrichment and risk stratification in specialized centres and clinical trials; currently less feasible for routine population-wide screening.
Urinary extracellular vesicles/urinary exosomes	The ISEV Urine Task Force and MISEV2023 provide consensus on technical aspects and reporting standards for urinary extracellular vesicles(uEVs) (78, 80). Clinical studies in diabetes and DKD have typically included tens to a few hundred participants. Saenz-Pipaon et al. analysed uEV protein content and reported that specific uEV markers can differentiate DKD from diabetes without nephropathy (79). Experimental and translational studies show that exosomes from patients with DKD can induce podocyte injury through miRNA-mediated mechanisms (82). Reviews describe the potential of uEVs as “liquid kidney biopsies” and summarize available clinical cohorts (77, 78).	Early-stage human studies suggest that uEV cargo reflects cell-type-specific damage and may detect podocyte and tubular injury before overt albuminuria or major eGFR decline, but current evidence is based on relatively small cohorts and surrogate outcomes (77–79, 82).	Non-invasive sampling; uEVs provide cell-type-enriched molecular information; high mechanistic relevance; compatible with multi-omic profiling and potentially suitable for serial monitoring.	Isolation, normalization and analysis methods are heterogeneous despite emerging standards (78, 80); small sample sizes; lack of large, endpoint-anchored prospective studies; no robust cost-effectiveness or implementation data.	Low to moderate – currently best regarded as a research and mechanistic tool; not yet ready for routine clinical risk stratification in DKD.
Multiparametric renal MRI and shear wave elastography (SWE)	Renal MRI techniques(diffusion-weighted imaging, BOLD MRI, arterial spin labelling)and functional MRI biomarkers have been evaluated in CKD and DKD cohorts ranging from ≈50 to 300 participants. PARENCHIMA consensus papers provide technical recommendations for clinical translation and standardization of renal MRI protocols (84–87). A prospective CKD cohort(n=305)showed that fMRI markers associate with baseline eGFR and predict subsequent eGFR decline (83). Systematic reviews and position papers summarize the role of MRI biomarkers in DKD trials and clinical practice (89, 90). SWE meta-analysis including >400 patients demonstrated that higher cortical stiffness correlates with biopsy-proven renal fibrosis (88).	Small to moderate-sized studies show that renal MRI and SWE parameters correlate with albuminuria, eGFR and histologic fibrosis, and may distinguish early from advanced kidney disease (83, 86–90). Consensus statements support technical feasibility and harmonization, but prognostic validation in large DKD-specific cohorts remains limited.	Direct, non-invasive assessment of renal perfusion, oxygenation, microstructure and stiffness; can detect tissue-level injury beyond conventional biomarkers; attractive as surrogate end points in trials and as an adjunct in complex cases.	High cost, limited availability, and need for specialized expertise; measurement variability across scanners and operators; absence of universally accepted thresholds; relatively few longitudinal, endpoint-anchored DKD studies.	Moderate – useful as an adjunctive tool in tertiary centres and for clinical trials; currently unsuitable as a first-line, population-wide screening modality.
Genetic and epigenetic markers; polygenic risk scores (PRS)	GWAS in T1D and T2D have identified multiple DKD-associated loci in cohorts of up to several thousand participants (24, 25). Epigenetic studies demonstrate that DNA methylation and histone modifications are linked to DKD and metabolic memory (23, 29, 31, 32, 36). Systematic reviews of genetic risk scores show that PRS can identify individuals at higher risk for DKD but often add modest predictive value over clinical models (27). Population-specific analyses(e.g., Hispanic/Latino cohorts and APOL1 high-risk genotypes)highlight ancestry-dependent performance of PRS for kidney traits (28, 30).	Genetic and epigenetic markers consistently associate with incidence and progression of DKD and help explain inter-individual susceptibility and metabolic memory (23–25, 27–32). However, their incremental prognostic value for short- to medium-term risk prediction beyond clinical factors is generally modest and varies by ancestry (27, 28, 30).	Provide stable lifetime risk indicators; offer mechanistic insights into pathways driving DKD; useful for stratifying participants in mechanistic and interventional studies; can support precision-medicine research.	Limited short-term clinical actionability; effect sizes for prediction are relatively small; performance varies across ancestries; integration into routine care requires careful consideration of equity, communication, and ethics (27–30, 32).	Low to moderate – mainly research and trial-enrichment tools at present; not yet recommended for routine DKD risk assessment in clinical practice.
TCM-derived digital phenotypes (tongue images, pulse/PPG signals)	Machine-learning analysis of tongue images has been applied to diabetes, metabolic disorders, MAFLD and DKD in cross-sectional and small longitudinal cohorts(typically n≈100–1,000). Studies include panoramic tongue imaging and deep CNN models for diabetes diagnosis (123), fusion of thermal and visible tongue images in diabetes (18), and standardized tongue-color acquisition with ICC profile correction (120). Tongue features have been compared between CKD and healthy participants (19)and used to develop diagnostic scales for TCM syndrome elements in DKD (116). Reviews summarize the current status of AI-based TCM tongue diagnosis and diagnostic models (115, 117, 124). Photoplethysmography-derived pulse wave and nocturnal pulse-wave attenuation have been linked to vascular ageing and cardiovascular risk (118, 119, 121), providing a framework for digital pulse assessment.	Proof-of-concept studies report moderate-to-high diagnostic performance for detecting diabetes or TCM patterns using tongue images (18, 115, 123, 124)and demonstrate differences in tongue features between CKD and healthy participants (19, 116). However, there are no large, endpoint-anchored cohorts directly linking TCM-derived digital phenotypes to hard DKD outcomes, and external validation across populations is scarce (115, 117, 126).	Low-cost, non-invasive, and well suited to high-frequency remote monitoring; culturally embedded in East Asian practice; can be combined with microbiome and other digital data to explore novel multimodal signatures (18, 116, 117, 126).	Image and signal acquisition are sensitive to device type, lighting, skin and mucosal pigmentation, and operator technique (120, 122). Methods and labels are heterogeneous; cross-cultural generalizability is uncertain; comparator studies versus established digital tools such as CGM and ABPM are lacking; regulatory and implementation pathways are not yet defined.	Low – currently exploratory and hypothesis-generating; should be confined to research settings and pilot digital-health programmes until rigorous standardization and validation are available.
Longitudinal machine-learning models integrating clinical data, biomarkers and digital signals	Longitudinal modelling of eGFR and uACR trajectories has been shown to improve prediction of kidney failure compared with single-timepoint models in CKD cohorts (91, 91, 96). Time-updated exposures and joint models are recommended for CKD research (97, 101–103). ML-based risk scores such as KidneyIntelX.dkd integrate biomarkers and EHR data to predict ≥40% eGFR decline or kidney failure and have been derived and validated in cohorts of several hundred to several thousand patients with T2D and early CKD (56–59). Other models using routinely collected EHR variables or minimal-resource predictors have been evaluated in diverse populations (94). CGM-derived metrics are associated with albuminuria, retinopathy and CKD in diabetes (99, 100, 113), and can be incorporated into dynamic risk-prediction frameworks.	Many longitudinal and ML models show good discrimination and calibration in internal and external validations, and time-updated modelling of kidney function and risk factors captures progression more accurately than baseline models (91, 91, 96). Integration of biomarker panels and digital data(e.g., CGM metrics)can further enhance risk stratification (56–59, 99, 100). A subset of models has reached regulatory review and real-world deployment(e.g., KidneyIntelX.dkd) (58, 59).	Able to leverage the full longitudinal history of eGFR, uACR and exposures; naturally aligns with KDIGO risk-based care and dynamic management; scalable within health systems using EHR data streams; can flexibly incorporate omics, imaging and digital phenotypes.	Vulnerable to dataset shift and calibration drift over time (110, 112); risk of bias and fairness concerns if training data are unrepresentative (109, 127); model updating and governance require explicit policies and infrastructure (91, 111, 112). Many models have not yet undergone prospective impact or cost-effectiveness evaluation.	Moderate to high – selected models are entering practice under regulatory oversight and can support individualized risk stratification; broader deployment requires robust governance frameworks, external validation in diverse settings, and demonstration of clinical and economic benefit (65–67, 93–95, 107, 114).

This comparative view underscores that many of the technologies discussed in this review are still largely hypothesis-generating. Only a subset have progressed to the level of clinically actionable tools that can be embedded into KDIGO 2024–aligned care pathways. Future research should therefore prioritize prospective, multi-center studies that benchmark novel markers directly against established anchors—eGFR, uACR, and KFRE—and report incremental value using calibration, reclassification, and clinical utility metrics.

## 4 Risk-driven therapeutic intensification and response monitoring

### 4.1 Foundational therapies: RAAS blockade and SGLT2 inhibitors

Early risk identification in DKD must be translated into the timely initiation of disease-modifying therapy. Across major guidelines, sodium–glucose cotransporter 2 inhibitors (SGLT2i) are now considered foundational for patients with CKD and albuminuria, regardless of diabetes status (5). Landmark trials such as DAPA-CKD and EMPA-KIDNEY confirmed consistent kidney-protective effects across diabetic and non-diabetic subgroups (6). These results support a risk-based rather than disease-centric approach, establishing SGLT2i as first-line agents when albuminuria is present and eGFR is within a safe prescribing range.

### 4.2 Add-on strategies for residual risk: non-steroidal MRAs and GLP-1 receptor agonists

For patients remaining at high risk—particularly those in the red/orange zones of eGFR-uACR grids or with persistent proteinuria—further intensification is warranted. Finerenone, a non-steroidal mineralocorticoid receptor antagonist (MRA), has demonstrated additive renal and cardiovascular benefit when added to background RAAS inhibition, as shown in FIDELIO-DKD and FIGARO-DKD (7). While hyperkalemia is a concern, it is manageable with serum potassium monitoring and stepwise titration. Recent data suggest that SGLT2i-MRA co-administration yields synergistic albuminuria reduction (128).

In parallel, glucagon-like peptide-1 receptor agonists (GLP-1RA) have shown renal benefit. The FLOW trial demonstrated that weekly semaglutide reduced the risk of composite kidney outcomes by 24%, alongside slowing eGFR decline and improving cardiovascular and all-cause mortality (12). These findings support GLP-1RA as a third-line agent for patients with persistent risk, obesity, or atherosclerotic disease despite optimized RAAS and SGLT2i therapy (129).

### 4.3 Monitoring efficacy and safety: risk-anchored protocols

KDIGO 2024 provides a framework for risk-anchored therapeutic monitoring. A modest eGFR drop (≤30%) following initiation of RAAS blockers or SGLT2i is expected and not grounds for discontinuation (130). However, greater declines necessitate evaluation of volume status, hypotensive burden, or drug–drug interactions. For finerenone, serum potassium and creatinine should be checked post-initiation and during titration (131). Reduction in UACR and flattening of eGFR slope are actionable intermediate endpoints, correlated with long-term outcome improvement, and suitable for outpatient decision-making.

### 4.4 A structured treatment pathway for DKD

A structured, risk-stratified treatment algorithm provides a pragmatic approach to therapeutic intensification in DKD. Initial risk classification can be achieved using eGFR–uACR heatmaps and the Kidney Failure Risk Equation (KFRE), enabling clinicians to identify patients requiring immediate intervention (5). For those with moderate to high risk, first-line therapy should combine SGLT2 inhibitors with optimized RAAS blockade (132). In cases of residual proteinuria or high total risk, non-steroidal MRAs such as finerenone may be added, followed by GLP-1 receptor agonists in patients with concurrent obesity, atherosclerotic disease, or inadequate risk control (133). Treatment effectiveness should be dynamically assessed using trends in UACR, eGFR slope, ambulatory blood pressure, and CGM-derived metrics (in diabetic individuals), with therapy adjusted accordingly. Importantly, transient eGFR declines post-initiation—particularly <30%—should not prompt discontinuation, but rather warrant evaluation of hemodynamic factors and reassessment of medication interactions to preserve long-term renoprotective benefit.

### 4.5 Compatibility with early detection and digital phenotyping tools

This risk-based therapeutic framework is inherently synergistic with the early detection technologies and TCM-informed digital phenotypes outlined in prior sections. Emerging tools such as multiparametric renal MRI, tongue/pulse signal analytics, and machine learning-based longitudinal prediction models can enhance the precision of risk assessment, allowing for earlier identification of high-risk individuals who may otherwise be missed using conventional markers alone. Moreover, continuous tracking of digital phenotypes and imaging biomarkers enables earlier detection of favorable treatment responses, providing a rationale for adaptive therapy escalation or de-escalation. Such integration supports more efficient resource allocation—targeting follow-up intensity and specialist referral toward those most likely to benefit—and reinforces a future-ready model of precision DKD management that bridges traditional and emerging paradigms.

### 4.6 Alignment with KDIGO 2024 and current care models

From a practical standpoint, only a subset of the technologies discussed in this review are currently ready for broad implementation in routine care. Within the KDIGO 2024 framework, annual or risk-based measurement of eGFR and uACR, combined with the eGFR×uACR heat map and KFRE, remains the mandatory foundation for DKD screening and risk stratification in primary care, endocrinology, and nephrology settings. Foundational therapies—optimized RAAS blockade, SGLT2 inhibitors, and, where appropriate, non-steroidal MRAs and GLP-1 receptor agonists—can be deployed today using existing guideline algorithms.

In contrast, urinary proteomics (CKD273), inflammatory/tubular biomarkers, multiparametric renal MRI, uEVs, and TCM-derived digital phenotypes are best viewed as adjunctive layers whose use should currently be confined to specialized centers, research protocols, or carefully designed implementation studies. When considered for clinical use, these tools should be embedded explicitly within guideline-based workflows—for example, as enrichment criteria for early nephrology referral, triggers for closer follow-up, or pharmacodynamic markers of therapy response—rather than as stand-alone replacements for established markers.

## 5 Ethics, regulation, and data governance

To enable the real-world implementation of multimodal early detection and risk-driven management frameworks, robust and replicable frameworks for ethics and data governance must be established from the outset. As modalities such as tongue imaging, pulse waveform acquisition, wearable monitoring, and home-based data collection extend data generation beyond clinical settings into daily life, the exposure surface for privacy risks increases substantially (134). Therefore, it is essential to adopt foundational data governance principles early in the research and deployment lifecycle. These include data minimization, purpose limitation, de-identification, and secure local storage. Furthermore, clear access controls and audit mechanisms must be established for data crossing national borders or interfacing with third-party algorithmic services.

When algorithms are integrated into clinical workflows as decision-support tools, human-machine collaboration must remain reversible and auditable (135). All automated outputs should include uncertainty indicators and interpretable insights, with the final clinical judgment retained by healthcare professionals (127). To ensure equitable utility, the model’s performance should be reported across subpopulations stratified by skin tone, language, health literacy, and device conditions. When disparities are observed, localized guidance and interface adaptations should be implemented to bridge the digital divide.

In addition to general principles of data protection, multimodal risk prediction systems for DKD often fall under the category of software as a medical device (SaMD). In the United States, the U.S. Food and Drug Administration (FDA) has published SaMD and Good Machine Learning Practice (GMLP) guidance documents that emphasize clear intended use, locked versus adaptive algorithms, predefined change-control plans, and ongoing post-market surveillance. Multimarker models such as KidneyIntelX.dkd provide an early example of a DKD-focused SaMD that has undergone *De Novo* review and authorization, highlighting the level of analytical and clinical validation expected for similar tools.

In Europe, AI-enabled diagnostic systems are subject to the Medical Device Regulation and the emerging European AI Act, which introduce risk-based classification, transparency requirements, and obligations for robustness, fairness, and human oversight. Systems used for high-stakes decisions in nephrology—such as predicting kidney failure or guiding treatment escalation—are likely to be categorized as “high-risk” AI, and therefore must undergo rigorous conformity assessment, documentation of training data provenance, and continuous performance monitoring.

At the level of data protection, region-specific frameworks such as the EU General Data Protection Regulation (GDPR), the U.S. Health Insurance Portability and Accountability Act (HIPAA), and national health data or personal information protection laws (e.g., China’s Personal Information Protection Law) mandate purpose limitation, data minimization, and strong safeguards for cross-border data transfer. For DKD-focused digital phenotyping, this implies that tongue images, pulse signals, and wearable data should be processed under explicit consent, with clear data retention policies and technical safeguards such as encryption, federated or on-device learning, and robust audit trails.

From a regulatory perspective, the evidence pathway for traditional biomarkers and imaging metrics typically progresses through three stages: analytical validity, clinical validity, and clinical utility. For software-as-a-medical-device (SaMD) systems, including multimodal risk prediction models, additional requirements apply (136). These include pre-specified protocols for change management, performance recalibration, and post-market surveillance. Regulatory approval and broad adoption are contingent upon a full chain of evidence demonstrating that the technology is safe, effective, interpretable, and economically viable. Only then can such integrated systems be ethically deployed and scaled in routine healthcare.

## 6 Limitations of current evidence and implementation considerations

Despite substantial progress, several limitations of the current evidence base must be acknowledged. First, many emerging biomarkers and imaging modalities have been evaluated primarily in small, single-center cohorts with limited ethnic diversity, short follow-up, and heterogeneous DKD phenotypes. For several technologies—particularly urinary extracellular vesicles, multiparametric renal MRI, shear wave elastography, and TCM-derived digital phenotypes—robust, multi-center studies anchored to hard endpoints (sustained eGFR decline, kidney failure, or cardio–renal composite outcomes) are still lacking.

Second, head-to-head comparisons between novel modalities and established markers are rare. As a result, it is often unclear whether a new test provides true incremental prognostic value beyond eGFR, uACR, and KFRE, or merely refines risk estimation in a statistically modest but clinically marginal way. Third, the majority of multimodal machine learning models have been developed and validated retrospectively in high-resource settings, with limited data on performance drift, equity, and generalizability in low- and middle-income countries.

Finally, health-economic and resource considerations are underexplored. High-throughput proteomics, multi-analyte biomarker panels, and advanced imaging techniques carry substantial per-patient costs and infrastructure requirements, which may not be feasible for routine use in many health systems. In contrast, simpler tools such as optimized eGFR and uACR measurement, structured risk scores, and guideline-directed pharmacotherapy are relatively inexpensive and widely implementable. Future research should therefore incorporate cost-effectiveness analyses and implementation science frameworks to determine which multimodal combinations deliver sufficient clinical benefit to justify real-world deployment.

## 7 Conclusion and future perspectives

The paradigm of DKD detection and management is undergoing a fundamental transformation—from static, late-stage biomarkers to an integrated, multimodal framework capable of capturing subclinical progression and guiding personalized intervention. Core to this evolution are validated anchors such as the eGFR×uACR grid and the Kidney Failure Risk Equation (KFRE), which now serve as the foundational layer upon which additional biological (e.g., blood and urine biomarkers), structural (e.g., functional renal imaging), and physiological (e.g., wearable-derived metrics) signals can be layered. Complementing these are digitally quantified TCM phenotypes—such as tongue features and pulse waveforms—which offer a low-cost, longitudinally accessible augmentation layer that captures subtle deviations from homeostasis.

Crucially, these technologies are not designed to replace current guideline-based workflows but to enhance them—particularly in the “gray zone” of early DKD where traditional indicators may be insensitive. When deployed within a risk-driven, closed-loop clinical pathway, these tools can shorten the lag between risk emergence and therapeutic intensification, enabling earlier and more targeted interventions. However, the translational focus must now shift from algorithmic discrimination to clinical impact. Future studies should prioritize hard outcomes such as sustained eGFR decline, ESKD, cardiovascular events, and health-economic metrics, underpinned by rigorous external validation across populations, settings, and acquisition devices.

Sustaining real-world utility will also require continuous model recalibration, data quality assurance, and bias mitigation strategies to ensure equitable performance across diverse subgroups. If implemented responsibly, this integrated and ethically grounded framework can deliver on the long-standing promise of precision nephrology: to identify high-risk individuals early, intervene proactively, and ultimately reduce the burden of DKD through individualized, scalable, and cost-effective care pathways.

## Glossary

ABPM: ambulatory blood pressure monitoring
AE: adverse event
AI: artificial intelligence
AKI: acute kidney injury
ASCVD: atherosclerotic cardiovascular disease
ASL: arterial spin labeling
AUC: area under the receiver-operating characteristic curve
BOLD: blood oxygen level–dependent
CGM: continuous glucose monitoring
CKD: chronic kidney disease
CKD-EPI: Chronic Kidney Disease Epidemiology Collaboration
CKD273: 273 urinary peptides
DKD: diabetic kidney disease
EHR: electronic health record
ERBP: European Renal Best Practice
ESKD: end-stage kidney disease
EV: extracellular vesicle
EVs: extracellular vesicles
FDA: U.S. Food and Drug Administration
GFR: glomerular filtration rate
GDPR: General Data Protection Regulation
GLP-1: glucagon-like peptide-1
GLP-1RA: glucagon-like peptide-1 receptor agonist
GMLP: Good Machine Learning Practice
GWAS: genome-wide association study
HbA1c: glycated hemoglobin A1c
HIPAA: Health Insurance Portability and Accountability Act
IDMS: isotope dilution mass spectrometry
KDIGO: Kidney Disease Improving Global Outcomes
KFRE: Kidney Failure Risk Equation
KIM-1: kidney injury molecule-1
LC-MS/MS: liquid chromatography–tandem mass spectrometry
MAFLD: metabolic dysfunction-associated fatty liver disease
MISEV2023: Minimal Information for Studies of Extracellular Vesicles 2023
ML: machine learning
MRA: mineralocorticoid receptor antagonist
MRI: magnetic resonance imaging
ns-MRA: non-steroidal mineralocorticoid receptor antagonist
PPG: photoplethysmography
PIPL: Personal Information Protection Law of the People’s Republic of China
PRS: polygenic risk score
PRIORITY: Proteomic prediction and Renin–Angiotensin system Inhibition prevention Of early diabetic nephRopathy In TYpe 2 diabetes trial
RAAS: renin–angiotensin–aldosterone system
RAS: renin–angiotensin system
RCT: randomized controlled trial
SaMD: software as a medical device
SGLT2: sodium–glucose cotransporter 2
SGLT2i: sodium–glucose cotransporter 2 inhibitor
SWE: shear wave elastography
T1D: type 1 diabetes
T2D: type 2 diabetes
TCM: Traditional Chinese Medicine
TNF: tumor necrosis factor
TNFR1: tumor necrosis factor receptor 1
TNFR2: tumor necrosis factor receptor 2
TRIPOD: Transparent Reporting of a multivariable prediction model for Individual Prognosis Or Diagnosis
eGFR: estimated glomerular filtration rate
ns-MRA: non-steroidal mineralocorticoid receptor antagonist
uACR: urine albumin-to-creatinine ratio
uEV: urinary extracellular vesicle
uEVs: urinary extracellular vesicles.
